# Autophagy Deficiency Induced by SAT1 Potentiates Tumor Progression in Triple‐Negative Breast Cancer

**DOI:** 10.1002/advs.202309903

**Published:** 2024-07-29

**Authors:** Wenwen Tian, Lewei Zhu, Yongzhou Luo, Yuhui Tang, Qingjian Tan, Yutian Zou, Kun Chen, Xinpei Deng, Hailin Tang, Hongsheng Li, Manbo Cai, Xiaoming Xie, Feng Ye

**Affiliations:** ^1^ State Key Laboratory of Oncology in South China Guangdong Provincial Clinical Research Center for Cancer Sun Yat‐sen University Cancer Center Guangzhou 510060 P. R. China; ^2^ Guangzhou Institute of Cancer Research, the Affiliated Cancer Hospital, Guangzhou Medical University Guangzhou 510095 P. R. China; ^3^ The First People's Hospital of Foshan Foshan 528000 P. R. China; ^4^ The First Affiliated Hospital Hengyang Medical School University of South China Hengyang Hunan 421001 P. R. China

**Keywords:** autophagy, m5C, SAT1, triple‐negative breast cancer, YBX1

## Abstract

Aggressive triple‐negative breast cancer (TNBC) still lacks approved targeted therapies, requiring more exploration of its underlying mechanisms. Previous studies have suggested a potential role of SAT1 (Spermidine/Spermine N1‐acetyltransferase 1) in cancer, which needs to be further elucidated in breast cancer. In this study, highly expressed SAT1 in TNBC signified worse patient prognoses. And SAT1 knockdown effectively inhibited the proliferation and migration abilities of TNBC cells in vitro and in vivo. In terms of mechanism, the transcription factor JUN enhanced SAT1 transcriptional activity by binding to its promoter region. Then, SAT1 protein in the cytoplasm engaged in directly binding with YBX1 for sustaining YBX1 protein stability via deubiquitylation mediated by the E3 ligase HERC5. Further, SAT1 was found to suppress autophagy remarkably via stabilization of mTOR mRNA with the accumulation of YBX1‐mediated methyl‐5‐cytosine (m5C) modification. These findings proved that SAT1 drives TNBC progression through the SAT1/YBX1/mTOR axis, which may provide a potential candidate for targeted therapy in advanced TNBC.

## Graphical abstract

Highly expressed SAT1 signifies worse prognoses in patients with TNBC. The transcription factor JUN enhances SAT1 transcriptional activity by binding to its promoter region. Subsequently, SAT1 stabilizes YBX1 protein through deubiquitylation and increases intracellular YBX1, which maintains the mRNA stability of mTOR through m5C modification to promote TNBC progression via inhibition of autophagy.

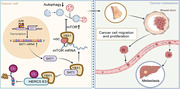

## 1 Introduction

As primary concern for female health worldwide, the survival outcomes of breast cancer patients differ significantly depending on molecular subtypes.^[^ ^1^, ^2^ ^]^ Owing to the absence of estrogen receptor (ER), progesterone receptor (PR), and human epidermal growth factor receptor 2 (HER2), 15–20% of breast cancers are characterized as triple negative breast cancers (TNBCs), which are the most aggressive subtype with the poorest prognosis.^[^ ^3^, ^4^ ^]^ Unravelling the molecular processes responsible for the development of TNBC remains a challenge, particularly to tailor‐made therapy for TNBC patients.

Located on the X chromosome, the SAT1 gene yields two transcripts: a 1085 bp mRNA of spermidine/spermine N1‐acetyltransferase 1 (SSAT1), which is a key metabolic modulator of polyamine metabolism; and a novel 1195 bp alternative splice variant that shows no coding potential but significant modifying activity of SSAT1.^[^ ^5^ ^]^ SSAT1 activity can be provoked by diverse factors, including cancer, despite maintaining typically subtle levels under normal conditions.^[^ ^6^ ^]^ The essential contribution of SAT1 dysfunction in cancer has been tentatively underlined in previous studies,^[^ ^7^ ^]^ including promoting radiotherapy resistance in glioblastoma^[^ ^8^ ^]^ and sensitizing cancer cells to undergo ferroptosis in response to reactive oxygen species (ROS)‐induced stress.^[^ ^9^ ^]^ Surprisingly, our earlier research has identified a population of tumor cells expressing SAT1 in breast cancer metastases,^[^ ^10^ ^]^ prompting us to delve deeper into the function of SAT1 in breast cancer.

The YBX family proteins are well defined by their capacity to bind to the Y box sequence (5′‐CTGATTGG‐3′) in DNA.^[^ ^11^ ^]^ Y‐box binding protein 1 (YBX1) expression is found in over 70% basal‐like breast cancers,^[^ ^12^ ^]^ and previous investigations have confirmed its oncogenic effects.^[^ ^13^, ^14^ ^]^ The 5‐methylcytosine (m5C) serves as an indispensable post‐transcriptional modification on mammalian mRNA, in which YBX1 stabilizes mRNA by binding directly to m5C‐methylated mRNA as a reader in the cytoplasm.^[^ ^15^, ^16^ ^]^ Recently, the function of m5C RNA modification in tumor development has received greater attention;^[^ ^17^, ^18^ ^]^ however, the emerging feature of m5C RNA modification in TNBC remains to be excavated.

The majority of eukaryotic cells sustain a basic level of autophagy, which has been defined as the process in which selected cellular contents are sequestered into double‐membrane vesicles to form phagosomes, followed by the fusion with lysosomes for degradation.^[^ ^19^ ^]^ Autophagy is known to operate in both tumorigenesis and anti‐tumor therapy response.^[^ ^20^ ^]^ In the initial stage of carcinogenesis, autophagy behaves as a tumor defender, a function that can be manifested, for example, by the fact that beclin‐1 deletion promoted the tumorigenesis of mammary epithelial cells developing tumors with TNBC features.^[^ ^21^ ^]^ Moreover, high expression of the autophagy gene ATG7 was found to restrain TNBC progression and induce TNBC cell apoptosis, which may contribute to improved prognosis and favorable clinicopathological characteristics in TNBC patients.^[^ ^22^ ^]^ Therefore, elucidating the molecular modulations of autophagy may uncover potential therapeutic targets for TNBC.

In this study, SAT1 was recognized as a tumor marker associated with unfavorable patient prognosis, and its amplification gave rise to a highly invasive phenotype in TNBC. Also, SAT1 was found to facilitate YBX1 protein stability by inhibiting HERC5‐mediated ubiquitination, which subsequently increased intracellular YBX1. Moreover, SAT1 could suppress autophagy through YBX1‐mediated m5C modification of mTOR mRNA. Thus, the current study has identified the SAT1/YBX1/mTOR axis as a neoplastic mechanism of TNBC.

## 2 Results

### 2.1 Upregulated SAT1 Imperils Survival Outcomes in TNBC Patients

**Figure 1:**
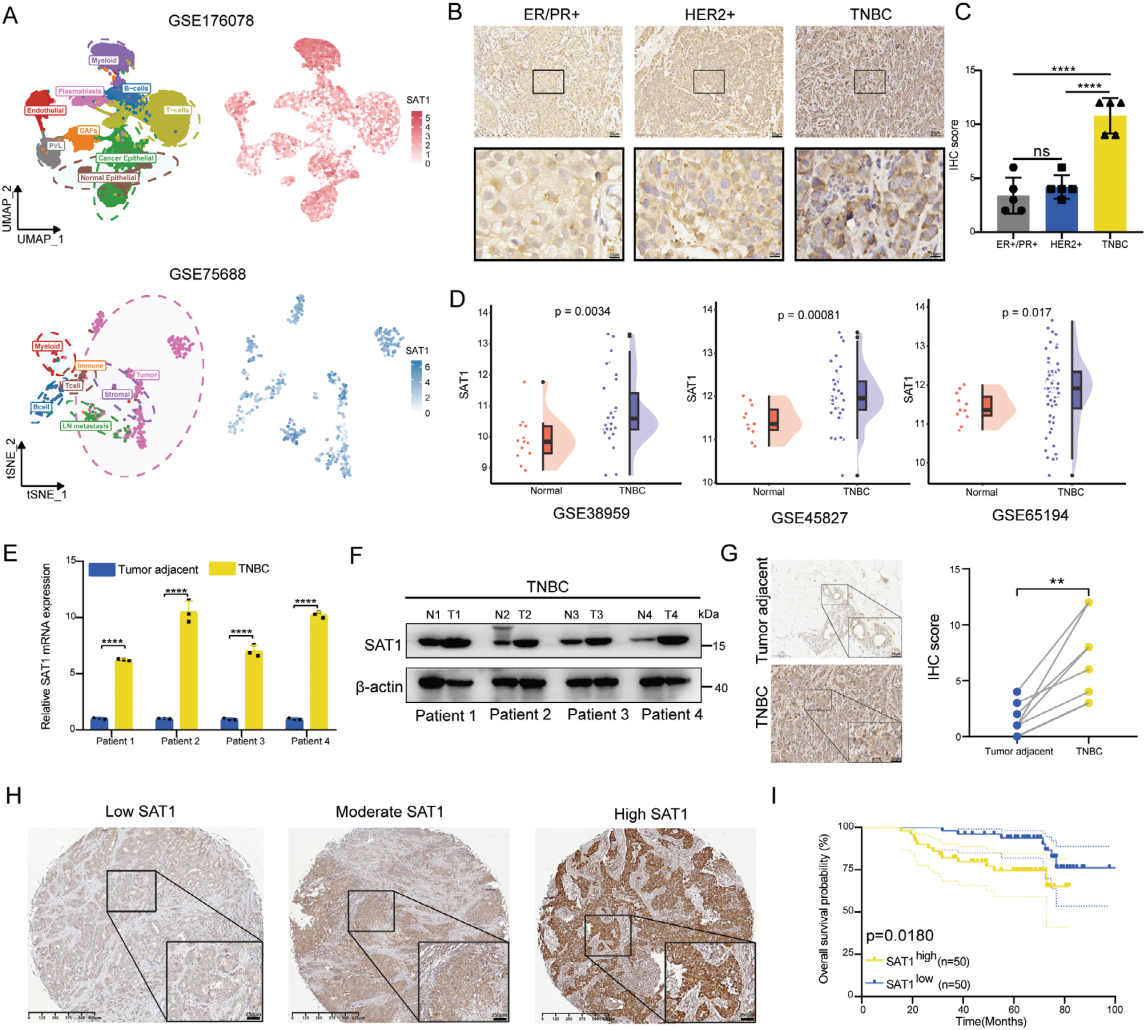
SAT1 expression is negatively correlated with TNBC prognosis. A) The UMAP plot in GSE176078 and t‐SNE plot in GSE75688 were performed to visualize cell types and SAT1 expression of each cell from breast cancer patients. B) The representative images of SAT1 expressions in different subtypes (n = 5) of breast cancer from SYSUCC using IHC. C) The bar plot of IHC scores in different subtypes of breast cancer from SYSUCC. D) Comparison of the SAT1 expression across normal tissues and TNBC tissues in GSE38959, GSE45827 and GSE65194. E–G) The SAT1 expressions were detected in paired tumor‐adjacent normal tissues and TNBC tissues (n = 4) through RT‐qPCR (E), western blots (F) and IHC (G). H,I) The representative IHC images (H) of various SAT1 expressions in TNBC and the Kaplan–Meier survival analysis (I) for OS performed in 100 TNBC patients based on different SAT1 levels.

In our previous research,^[^ ^10^ ^]^ a subset of SAT1‐labeled cells was identified in breast cancer metastases, however, very limited evidence of SAT1 regarding to breast cancer has been reported. To investigate this, firstly, the analyses of two single‐cell sequencing cohorts (GSE176078 and GSE75688) revealed that SAT1 may serve as a marker gene for certain cell populations in breast cancer (**Figure** **1**A). Then, the CytoTRACE analysis in GSE176078 cohort revealed that breast cancer cells with high SAT1 expressions have a higher prevalence of cancer cell stemness (Figure S1A,B, Supporting Information), which also implied that SAT1 may facilitate tumor progression. To confirm this, we first examined the presence of SAT1 in different molecular subtypes of breast cancer, and found that SAT1 was extremely abundant in TNBC, compared to ER/PR‐positive or HER2‐positive breast cancers (Figure 1B,C). The expressions of SAT1 in three TNBC datasets (GSE38959, GSE45827, and GSE65194) were found to be significantly upregulated in tumors comparing to adjacent normal tissues (Figure 1D), which was reconfirmed in patient‐derived TNBC samples from SYSUCC, both at the mRNA and protein levels (Figure 1E–G). Then, the Kaplan–Meier survival analyses for OS (Figure S1C, Supporting Information) and RFS (Figure S1D, Supporting Information) in the TCGA‐BRCA cohort demonstrated that SAT1 upregulation reduced survival probabilities of TNBC patients rather than non‐TNBC patients. Further, the tissue microarray containing tumor specimens from 100 TNBC patients also documented that elevated SAT1 adversely affected patient survival outcomes regardless of its varied expressions (Figure 1H,I).

### 2.2 Highly Expressed SAT1 Potentiates TNBC Progression In Vitro and In Vivo

Since SAT1 upregulation was related to inferior prognoses of TNBC patients, it should also have a profound effect on tumor behaviors. Through RT‐qPCR (Figure S2A, Supporting Information) and western blots (Figure S2B, Supporting Information), we first determined SAT1 expression in multiple TNBC cell lines, indicating strong expressions in BT549 and SUM159PT cells and relatively weak expression in MDA‐MB‐231 cells. Using the lentiviral packaging system, SAT1 was stably knocked down in BT549 and SUM159PT cells (Figure S2C,D, Supporting Information) while overexpressed in MDA‐MB‐231 cells (Figure S2E,F, Supporting Information). The CCK8 (Figure S2G, Supporting Information) and clone formation assays (Figure S2H, Supporting Information) confirmed that SAT1 stimulated TNBC cell proliferation efficiently in vitro. Similarly, upregulated SAT1 enhanced TNBC cell migration in scratch wound healing (Figure S2I, Supporting Information) and transwell migration experiments in vitro (Figure S2J, Supporting Information). Since epithelial‐mesenchymal transition (EMT) has been characterized as a remarkable hallmark of cancer progression and metastasis,^[^ ^23^, ^24^ ^]^ EMT‐related proteins were also found to be inactivated in response to SAT1 knockdown (Figure S1K, Supporting Information). The above experimental data in vitro established the tumor‐promoting capacity of SAT1 in TNBC.

**Figure 2:**
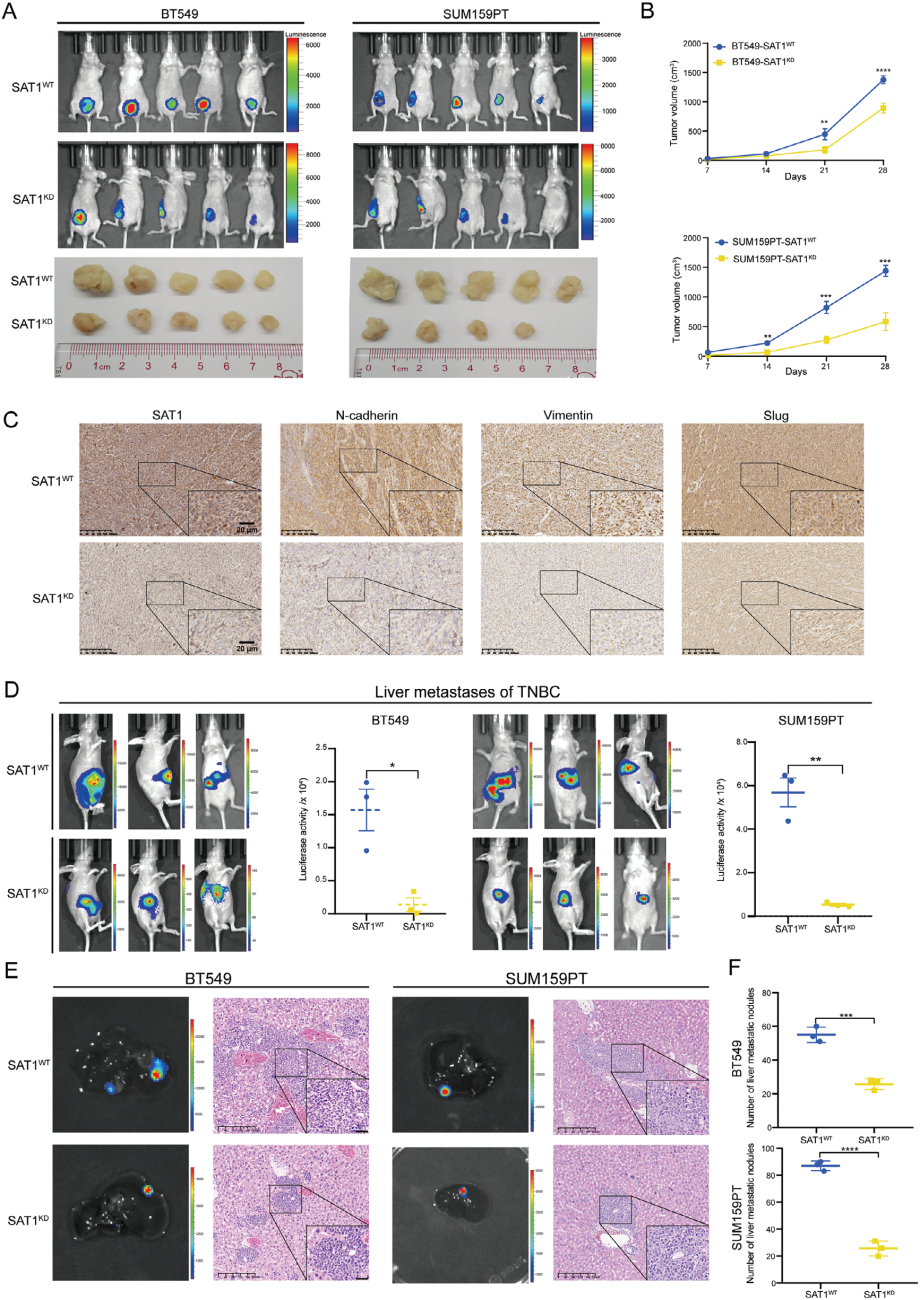
SAT1 knockdown inhibits TNBC progression in vivo. A) The in vivo bioluminescence imaging and tumor images of xenograft models generated by SAT1^WT^ and SAT1^KD^ cells (n = 5). B) The tumor growth curves of mice xenograft models generated by SAT1^WT^ and SAT1^KD^ cells. C) IHC staining of EMT‐related markers in tumor tissues derived from mice xenograft models in both SAT1^WT^ and SAT1^KD^ groups. D) The in vivo bioluminescence imaging (left) and luciferase activity analysis (right) of mice liver metastases models in both SAT1^WT^ and SAT1^KD^ groups (n = 3). E,F) The bioluminescence images and HE staining of mice liver metastases (E), and statistics on the number of liver metastatic nodules (F) in both SAT1^WT^ and SAT1^KD^ groups.

To determine the vital function of SAT1 in vivo, we developed subcutaneous xenograft and liver metastasis models in female BALB/c nude mice, respectively. As observed from the xenograft model, tumors developed by SAT1^KD^ cells exhibited a significant reduction in tumor volume (**Figure** **2**A), which also manifested in reduced tumor growth (Figure 2B). The expression levels of EMT‐associated proteins in tumors retrieved from the SAT1^KD^ xenograft model also declined as expected (Figure 2C). At 6 weeks after establishing liver metastasis models successfully, in vivo bioluminescence imaging revealed diminished liver metastases in the SAT1^KD^ group (Figure 2D). And in HE‐stained liver tissues from metastasis models, we also observed fewer and smaller metastatic nodules in the SAT1^KD^ group (Figure 2E). The above conclusions laid the cornerstone for our subsequent research.

### 2.3 JUN Enhances SAT1 Transcription Activity Through Binding to the SAT1 Promoter

**Figure 3:**
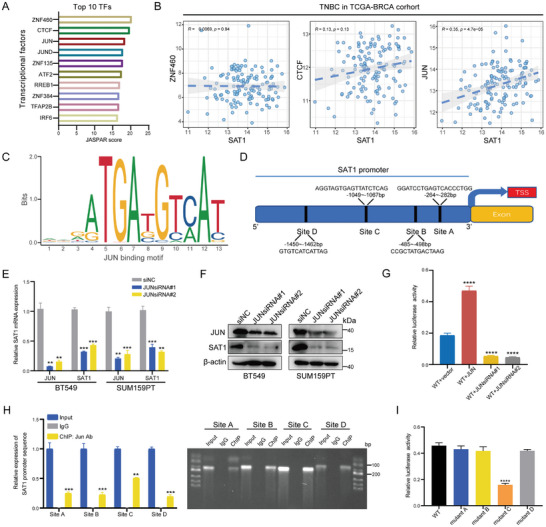
JUN boosts SAT1 transcription by binding to its promoter sequence. A) The top 10 transcription factors (TFs) binding to SAT1 promoter were obtained from the JASPAR database. B) The relationship between SAT1 expression and the top three transcription factors was determined with Spearman's correlation analyses in TNBC samples from TCGA‐BRCA cohort. C) The binding motif of JUN on SAT1 promoter. D) Four potential sites where JUN binds to SAT1 promoter were obtained from the JASPAR database. E,F) The alterations of SAT1 in response to JUN inhibition were detected by RT‐qPCR (E) and western blots (F), respectively. G) The dual‐luciferase reporter assay was used to confirm the binding of JUN to SAT1 promoter. H) The ChIP assay with JUN antibody was employed to verify the direct binding of JUN to SAT1 promoter. I) The dual‐luciferase reporter assay reaffirmed the key binding site of JUN to SAT1 promoter sequence.

Due to the significance of SAT1 upregulation in TNBC, we investigated its upstream transcription factor, which is essential in regulating the coding gene expression. Targeting the SAT1 promoter sequence, we obtained ten transcription factors from the JASPAR database (**Figure** **3**A).^[^ ^25^ ^]^ Among three transcription factors with the highest matched scores, JUN was found to have the most robust positive correlation with SAT1, implying that JUN has the greatest potential to modulate SAT1 expression at the transcriptional level (Figure 3B). Of the 13 predicted sites where JUN binds to the SAT1 promoter, we identified the four most probable binding sites for validation through JASPAR database (Site A: GGATCCTGAGTCACCCTGG; site B: CCGCTATGACTAAG; site C: AGGTAGTGAGTTATCTCAG; site D: GTGTCATCATTAG; Figure 3C,D). Upon siRNA silence of JUN in indicated cells, SAT1 exhibited obvious downregulation in both mRNA (Figure 3E) and protein levels (Figure 3F). After cloning the full‐length SAT1 promoter sequence into a dual‐luciferase vector, the dual‐luciferase reporter assay revealed that their binding was weakened with JUN knockdown as well as enhanced with JUN overexpression (Figure 3G). In an effort to determine the exact binding site between JUN and SAT1 promoter, the ChIP assay using a JUN antibody confirmed that JUN bound directly to varying degree to all four sites of the SAT1 promoter sequence (Figure 3H). The deletion mutants of the four sites were similarly generated in dual‐luciferase vectors, which were transfected into BT549 cells. Dual‐luciferase reporter assays demonstrated that the deletion mutant targeting site C mainly affected the binding of JUN and SAT1 promoter region, suggesting that site C dominated for JUN to enhance SAT1 transcriptional activity (Figure 3I).

### 2.4 YBX1 Mediates the Function of SAT1 in Contributing to TNBC Progression

**Figure 4:**
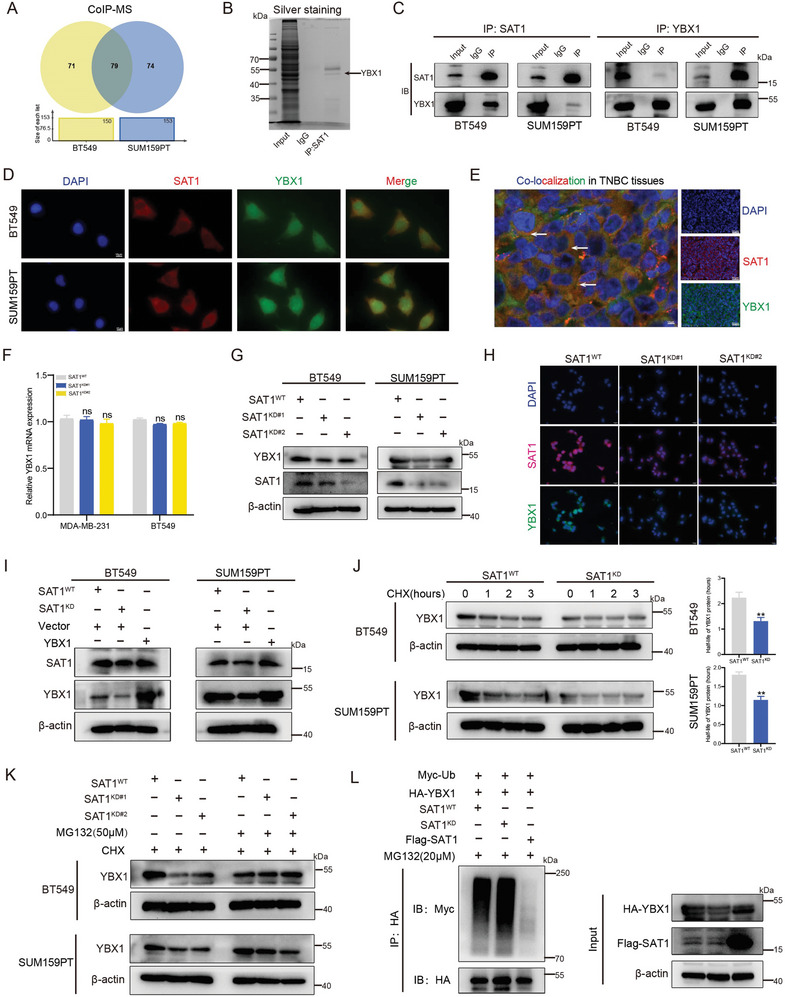
SAT1 stabilizes YBX1 protein through deubiquitylation. A) Identification of 79 interacted proteins of SAT1 through CoIP‐MS analyses in both BT549 and SUM159PT cells. B) The silver staining after immunoblotting of SAT1 in BT549 cells. C) The interaction between SAT1 and YBX1 was verified using CoIP followed by immunoblotting. D,E) Co‐localizations of SAT1 and YBX1 in both TNBC cells (D) and tissues (E) were visualized with immunofluorescence. F) The RT‐qPCR for YBX1 mRNA expression in SAT1^WT^ and SAT1^KD^ cells. G) Western blots for YBX1 protein expression in SAT1^WT^ and SAT1^KD^ cells. H) Immunofluorescence images for YBX1 protein in SAT1^WT^ and SAT1^KD^ cells. I) Western blots for SAT1 in SAT1^KD^ cells with or without YBX1 overexpression. J) Western blots for YBX1 at different times (left) and quantization of half‐life time (right) of YBX1 in SAT1^WT^ and SAT1^KD^ cells in response to CHX (50 µm) treatment. K) Western blots for YBX1 in SAT1^WT^ and SAT1^KD^ cells after MG132 (50 µm) treatment for 12 h. L) Immunoblots of cell lysate and HA‐tagged immunoprecipitants from HEK293T cells transfected with the indicated plasmids to detect the alteration of HA‐YBX1 ubiquitylation level.

To elucidate how SAT1 facilitates TNBC progression, we recognized 79 interacting proteins from CoIP‐MS analyses in both BT549 and SUM159PT cells (**Figure** **4**A). Among these proteins, YBX1 was found to be most highly expressed in the TNBC subtype of breast cancer (Figure S3A, Supporting Information), and highly expressed YBX1 significantly affected the OS and RFS survival probabilities of breast cancer patients (Figure S3B, Supporting Information). A clear band matching the protein size of YBX1 was also detected on the silver‐stained gel (Figure 4B). The above findings suggested to us that SAT1 action might be fueled via interacting with YBX1. Shown in Figure 4C, the binding between SAT1 and YBX1 was verified in both BT549 and SUM159PT cells. And co‐localization of SAT1 and YBX1 was observed in both TNBC cells (Figure 4D) and patient‐derived tissues (Figure 4E). Following SAT1 knockdown, alteration of YBX1 occurred considerably at the protein level (Figure 4G,H) rather than the mRNA level (Figure 4F). We further found that YBX1 expression declined in mice model‐derived tissues of the SAT1^KD^ group (Figure S3C, Supporting Information). With no change of SAT1 protein was observed following YBX1 re‐overexpression in SAT1^WT^ cells, YBX1 was determined as the downstream of SAT1 (Figure 4I). The rescue experiments in vitro demonstrated that both the inhibition of cell proliferation (Figure S3D, Supporting Information) and migration in SAT1^KD^ cells could be restored by YBX1 amplification (Figure S3E, Supporting Information). The same finding was noted in the detection of EMT‐related proteins (Figure S3F, Supporting Information). Thus, we concluded that the function of SAT1 to facilitate TNBC progression was modulated by YBX1.

### 2.5 SAT1 Stabilizes YBX1 Through Deubiquitylation Mediated by the E3 Ligase HERC5

Subsequently, treatment with CHX (50 µM) augmented the decrease of YBX1 in SAT1^KD^ cells (Figure 4J), whereas MG132 (50 µM) treatment increased the level of YBX1 protein (Figure 4K). The ubiquitination level of YBX1 protein increased obviously with the decrease of SAT1 (Figure 4L), demonstrating that upregulated SAT1 prevented YBX1 from undergoing ubiquitin‐mediated degradation.

**Figure 5:**
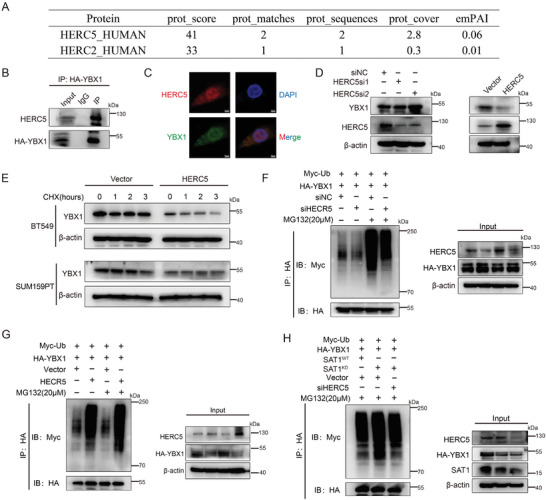
HERC5 mediates YBX1 ubiquitination induced by SAT1 knockdown. A) The protein mass spectrometry data of YBX1 immunoprecipitants from BT549 cells. B) Co‐immunoprecipitation for indicated protein followed by immunoblots for HERC5 in BT549 cells. C) Co‐localization of HERC5 and YBX1 in BT549 cells was visualized with immunofluorescence. D) Western blots for YBX1 in BT549 cells with HERC5 inhibition or overexpression. E) Western blots for YBX1 in TNBC cells with overexpressed HERC5 in response to CHX (50 µM) treatment for different periods. F,G) Immunoblots of cell lysate and HA‐tagged immunoprecipitants from HEK293T cells with HERC5 inhibition (F) or overexpression (G). H) Immunoblots of cell lysate and HA‐tagged immunoprecipitants from HEK293T cells with inhibition of both SAT1 and HERC5.

Going a step further to understand how SAT1 maintains YBX1 protein stability through deubiquitylation, we investigated the mass spectrometry data of YBX1 protein and found two E3 ligases, including HERC2 and HERC5 (**Figure** **5**A). Since HERC5 possessed higher abundance and more matching peptides, it was considered as the main E3 ligase to achieve YBX1 ubiquitination. HERC5 was verified to be capable of binding to YBX1 through CoIP assay (Figure 5B) and cellular co‐localization (Figure 5C). Increased HERC5 resulted in a dramatic decrease of YBX1 protein along with a slight elevation of YBX1 protein following HERC5 inhibition (Figure 5D). With CHX intervention, more apparent degradation of the YBX1 protein was observed in TNBC cells with upregulated HERC5 (Figure 5E). The ubiquitination assays indicated that ubiquitinated degradation of YBX1 protein was subsequently attenuated by HERC5 knockdown (Figure 5F), and conversely this process was accelerated by HERC5 overexpression (Figure 5G). Moreover, the enhanced ubiquitination level of YBX1 protein eliciting in SAT1^KD^ cells could be reversed successfully by abating HECR5 (Figure 5H). These results suggested that SAT1 could stabilize YBX1 protein through HERC5 mediation.

### 2.6 Autophagy Depression Induced by SAT1/YBX1 Fosters TNBC Progression

**Figure 6:**
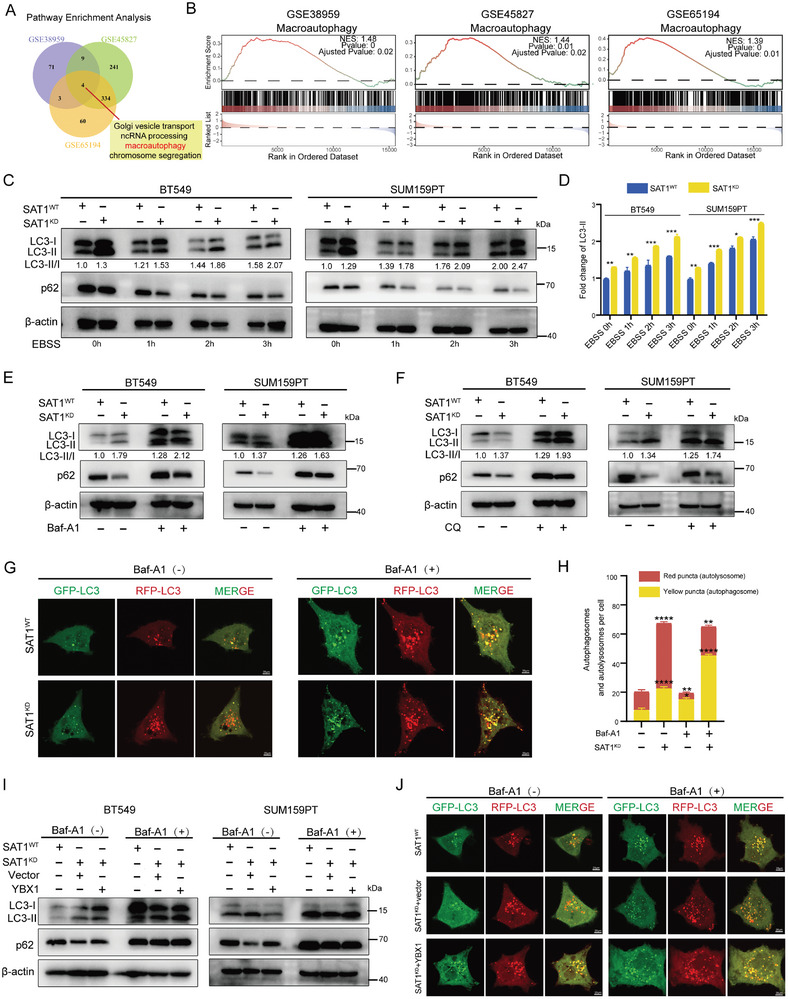
SAT1 deficiency activates autophagy in TNBC. A) The Venn diagram showed pathway enrichment analyses of SAT1 in three TNBC cohorts (GSE38959, GSE45827 and GSE65194). B) The GSEA results of autophagy in the above three datasets. C,D) Western blots for LC3 and p62 in SAT1^WT^ or SAT1^KD^ cells after EBSS treatment for indicated hours (C); The fold change of LC3 II was calculated by bar plot (D). E) Western blots for LC3 and p62 in SAT1^WT^ or SAT1^KD^ cells with or without BafA1 (100 nm) treatment for 12 h. F) Western blots for LC3 and p62 in indicated cells with or without chloroquine treatment for 12 h. G,H) SAT1^WT^ and SAT1^KD^ BT549 cells transfected with an mRFP‐GFP‐LC3 reporter were treated with or without BafA1 (100 nm) for 12 h. Representative confocal images were presented G), and the number of autophagosomes (yellow puncta) and autolysosomes (red puncta) per cell was quantified H). I) Western blots for LC3 and p62 in SAT1^KD^ cells with or without Baf‐A1 (100 nM) treatment in response to YBX1 overexpression. J) The representative confocal images of mRFP‐GFP‐LC3 assay in SAT1^WT^ and SAT1^KD^ BT549 cells which were transfected with or without YBX1 overexpression and treated with or without Baf‐A1 (100 nM) for 12 h.

By performing GSEA analyses of the transcriptome matrix from multiple TNBC cohorts (GSE38959, GSE45827, and GSE65194), we found that SAT1 was strongly involved in macroautophagy (**Figure** **6**A,B). In spite of the bipartite role of autophagy in cancer,^[^ ^26^, ^27^ ^]^ it remains uncertain that how autophagy functions in TNBC. In the presence of the autophagy inducer EBSS, the LC3‐II/I protein level in SAT1^KD^ cells obviously elevated compared to SAT1^WT^ cells, while p62 declined more apparently compared to control cells (Figure 6C,D). Meanwhile, increased LC3‐II/I and p62 were observed after dealing SAT1^KD^ cells with the lysosomal inhibitors Baf‐A1 (Figure 6E) and CQ (Figure 6F). To further observe the autophagy flux, we found that SAT1 inhibition increased both red and yellow puncta in BT549 cells stably transfected with GFP‐mRFP‐LC3, while treatment with Baf‐A1 reduced red puncta (autolysosomes) as well as increased yellow puncta (autophagosomes) (Figure 6G,H). This finding demonstrated the activation of autophagy in SAT1^KD^ cells, which was further validated in mice model‐derived tumor tissues (Figure S4A, Supporting Information).

Further, we detected the proliferation and migration abilities of SAT1^KD^ cells after treatment with Baf‐A1, proving that SAT1 knockdown limited TNBC advancement by activating autophagy (Figure S4B,C, Supporting Information). Since both YBX1 and autophagy deficiency were responsible for TNBC progression induced by SAT1, we reasoned that there should be an identifiable relationship between them. Some studies has suggested that YBX1 participates in the regulation of autophagy.^[^ ^28^, ^29^ ^]^ Not surprisingly, recovered expression of YBX1 in SAT1^KD^ cells was capable of lowering the LC3‐II/I level and raising the p62 level, implying that cell autophagy was reverted to a suppressed condition (Figure 6I). This effect was also observed in autophagy flux. As shown in Figure 6J, diminished red puncta resulted by the presence of YBX1 strengthened additionally with Baf‐A1 treatment. Namely, YBX1 did interfere with the regulation of autophagy arising from SAT1.

It is worth mentioning that autophagy deficiency in TNBC has been reported to inhibit T cell‐mediated tumor killing, which subsequently impaired immunotherapy response.^[^ ^30^ ^]^ Therefore, we obtained public immunotherapy data from IMvigor210 to evaluate the relationship between SAT1 and immunotherapy response. From the correlation analyses in TNBC samples of TCGA‐BRCA and GSE96058, a positive correlation was observed between SAT1 and PD1 as well as PD‐L1 (Figure S4D, Supporting Information). The expression boxplots in TCGA‐TNBC and IMvigor210 datasets also exhibited higher PD1 and PD‐L1 in high SAT1 group (Figure S4E, Supporting Information). This surprising finding was further verified in TNBC tissues using IHC (Figure S4F, Supporting Information), which suggested a potential association between SAT1 and the immune microenvironment of TNBC.

### 2.7 SAT1/YBX1 Enhances the mRNA Stability of mTOR via m5C Modification

**Figure 7:**
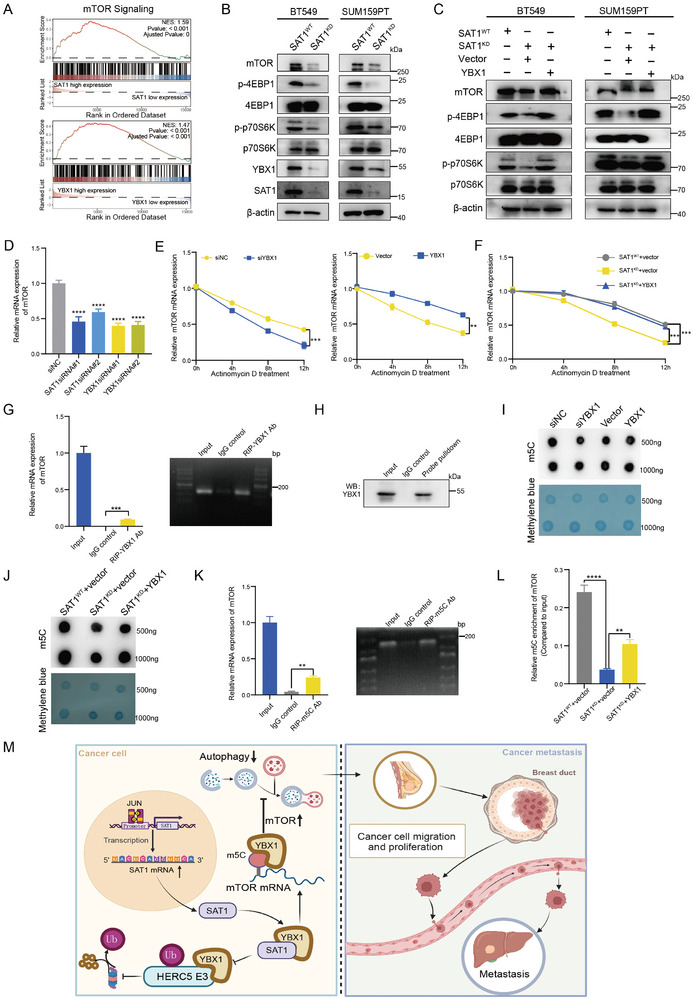
SAT1 suppresses autophagy through YBX1‐mediated m5C modification of mTOR mRNA. A) The GSEA results of mTOR signaling pathway in a TNBC cohort‐GSE38959. B) Western blots for mTOR and its downstream effectors in SAT1^WT^ and SAT1^KD^ cells. C) Western blots for mTOR and its downstream effectors in SAT1^KD^ cells with or without overexpressed YBX1. D) qPCR for the mTOR mRNA expression in indicated cells with knockdown of SAT1 or YBX1. E) mRNA quantification of mTOR in BT549 cells with YBX1 knockdown or overexpression in response to actinomycin D treatment for indicated hours. F) mRNA quantification of mTOR in SAT1^WT^ and SAT1^KD^ BT549 cells with or without YBX1 overexpression in response to actinomycin D treatment. G) The RIP‐qPCR assay to detect the binding between YBX1 and mTOR mRNA in BT549 cells. H) Lysates of BT549 cells were pulled down with biotinylated probe recognizing mTOR mRNA, and then YBX1 in the precipitates was detected by western blots. I) Dot blot assay for m5C levels in BT549 cells with YBX1 knockdown or overexpression. The intensity of dot immunoblotting (above) represented the m5C levels while methylene blue staining (below) indicated the amount of loaded RNA. J) Dot blot assay for m5C levels in SAT1^WT^ and SAT1^KD^ BT549 cells with or without YBX1 overexpression. K) The RIP‐qPCR assay to verify the binding between m5C and mTOR mRNA in BT549 cells. L) The RIP‐qPCR assay to qualify m5C enrichment levels of mTOR mRNA in SAT1^WT^ and SAT1^KD^ BT549 cells with or without YBX1 overexpression. (M) A schematic of the conclusion in this current study.

As a gatekeeper, the mTOR signaling pathway has been the central for limiting the activation of autophagy.^[^ ^31^ ^]^ Therefore, we hypothesized that the suppressive autophagy exhibited by SAT1/YBX1 complex would most likely be mediated via the mTOR signaling pathway. First, the GSEA analysis in earlier TNBC dataset (GSE38959) revealed that both SAT1 and YBX1 were positively relevant to mTOR signaling, respectively (**Figure** **7**A). A significant decrease of mTOR and downstream molecules occurred in SAT1^KD^ cells (Figure 7B), which could be restored by YBX1 overexpression (Figure 7C). The RT‐qPCR analysis indicated that both reduced SAT1 and YBX1 elicited remarkable curbing of the mTOR transcription level, prompting us that the SAT1/YBX1 complex was competent to monitor mTOR at its transcriptional level (Figure 7D). Consequently, we examined whether the mRNA stability of mTOR would be impacted upon SAT1 or YBX1 using actinomycin D. With extended exposure to actinomycin D, the quantity of mTOR mRNA declined at greater levels in BT549 cells with YBX1 silencing and, conversely, accumulated more heavily in cells with overexpressed YBX1 (Figure 7E). More impressively, the reduced mTOR mRNA entailed in SAT1^KD^ BT549 cells could be reinstated by YBX1 overexpression (Figure 7F). These data signified that SAT1 maintained the mRNA stability of mTOR through YBX1 mediation in TNBC.

Notably, recent studies have identified YBX1 as a cytoplasmic reader of mRNA m5C modification, whose prevalence provides a pivotal function in the regulation of RNA metabolism.^[^ ^32^, ^33^ ^]^ We reasoned that the SAT1/YBX1 complex may exert m5C modification on mTOR mRNA mediated by YBX1, consequently leading to autophagy repression. First, the RIP assay by YBX1 antibody showed a direct binding action of YBX1 protein to the mTOR mRNA in BT549 cells (Figure 7G). Consistently, YBX1 was pulled down by biotinylated probe specifically recognizing mTOR mRNA (Figure 7H). The dot blot assay revealed that YBX1 inhibition led to a lower m5C level while overexpressed YBX1 led to a higher m5C level (Figure 7I). We also observed that the decreased m5C level in SAT1^KD^ cells could be rescued by YBX1 overexpression, which implied that the mRNA stabilization of mTOR regulated by the SAT1/YBX1 axis was dominated by m5C modification (Figure 7J). A higher content of an mRNA from the isolated RNA means a higher m5C level of the mRNA. From the RIP assays, it was clear that the mTOR mRNA was not only detectable for binding to the m5C antibody (Figure 7K), but also their attenuated incorporation resulted from SAT1 downregulation could be reversed by YBX1 (Figure 7L). Based on these results, we concluded that SAT1 inhibits autophagy to favor TNBC progression through YBX1‐mediated mRNA sustainment of mTOR via m5C modification (Figure 7M).

## 3 Discussion

Both experimental and clinical evidence in our study suggested a potentially interesting mechanism of tumor progression in TNBC. We identified that highly expressed SAT1 in TNBC is linked to worse patient survival, accounting for the autophagy‐deficient condition of TNBC. In this mechanism, the transcription factor JUN binds to the SAT1 promoter region to boost the SAT1 transcriptional activation. Then, SAT1 protein in the cytoplasm engages in direct binding with YBX1 for sustaining YBX1 protein stability. SAT1 enables the deubiquitylation of YBX1, which is mediated by the E3 ligase HERC5. Then, increased cytoplasmic YBX1 serves as an m5C reader binding to the mRNA of mTOR and maintaining its mRNA stability via m5C modification. Finally, SAT1 activates the negative regulatory mechanism of autophagy through stabilizing the mTOR mRNA, leading to cell proliferation and metastasis of TNBC. These observations suggested that SAT1 could be a therapeutic candidate of targeted therapy strategies for TNBC patients.

Although some studies have illustrated that knockout of SAT1 expression partially eliminates p53‐mediated ferroptosis,^[^ ^9^ ^]^ others have revealed that upregulated SAT1 expression in tumors is strongly correlated with adverse outcomes of cancer patients.^[^ ^34^, ^35^, ^36^ ^]^ We reconfirmed this conclusion in TNBC with both in vivo and in vitro experiments. SAT1 knockdown in TNBC cells could restrain cell proliferation and migration in vitro, as well as retard in vivo tumor growth at the primary site and the development of distant liver metastases. It is our intention in the future to examine whether spontaneous tumorigenesis in transgenic murine models will be affected by knocking out SAT1.

The interaction between tumor progression and autophagy are complicated. Autophagy has been proven to be an indispensable cellular function that has consequently attracted great interest in cancer research.^[^ ^19^, ^37^ ^]^ The majority of cells uphold a fundamental level of autophagy which serves for homeostasis, removal of damaged organelles, and nutrient cycling.^[^ ^38^ ^]^ Early evidence hinted at the contribution of autophagy‐related proteins in mediating tumorigenesis, both positively and negatively, and autophagy often takes on varied roles during different phases of tumorigenesis.^[^ ^39^, ^40^ ^]^ Thus, the modulation of autophagy participates in cancer development depends on the individual characteristics of specific molecules, including SAT1. In this study, we validated that defective autophagy induced by SAT1 promoted TNBC progression, which is consistent with previous conclusions that the autophagic capacity of breast cancer cells, especially TNBC tumors, may be impaired at basal levels or after exposure to various stresses.^[^ ^41^, ^42^, ^43^ ^]^ In addition, autophagy‐deficient TNBC cells were found to trigger mTOR mRNA stabilization enabled by m5C modification, which was completed by YBX1. YBX1 is overexpressed in aggressive tumors, acting as an oncogene,^[^ ^44^, ^45^, ^46^ ^]^ including breast cancer.^[^ ^47^, ^48^ ^]^ Notably, YBX1 displayed functional importance in autophagy through directly targeting m5C‐containing Ulk1 mRNA or inhibiting the p110β/Vps34/beclin1 signaling pathway.^[^ ^49^ ^]^ Since YBX1 induced autophagy in other cancers, it showed the opposite effect in TNBC. We suggested that the reason lies not only in the diversity of cancer types, but also in the molecular pathways through which YBX1 acts in autophagy. Of greater importance, the elucidation of autophagy in TNBC warrants more investigation.

It is noteworthy that our study preliminarily suggested the relevance of SAT1 to immunotherapy through the regulation of autophagy. It is widely acknowledged that autophagy plays a role in immunity, and autophagy has lately been involved in cancer immunotherapy.^[^ ^50^ ^]^ Since cancer cells can evade immune detection by inhibiting autophagy, autophagy boosters may improve the efficacy of cancer immunotherapy.^[^ ^51^ ^]^ In this study, we observed a positive correlation between SAT1 and PD1 as well as PD‐L1, which hinted that TNBC patients with high SAT1 expression may respond better to immunotherapy. However, high SAT1 expression is accompanied by autophagy deficiency, which is contradictory to the findings of previous studies. It is essential to explore intensively the effect of SAT1 on immunotherapy in future studies.

## 4 Conclusion

In summary, this study identified SAT1 as a potential candidate for targeted therapy strategies in TNBC patients. And upregulated SAT1 in TNBC was verified to facilitate tumor progression through the SAT1/YBX1/mTOR axis.

## 5 Experimental Section

### Patient Sample Collection

This study was approved by Institutional Research Ethics Committee of Sun Yat‐sen University Cancer Center (SYSUCC) and conducted according to the guidance of the Declaration of Helsinki. Breast cancer samples were obtained from postoperative specimens of patients diagnosed with breast cancer. Breast tumor tissue cores from 100 TNBC patients were collected to construct a microarray for subsequent validation and patients without recorded follow‐up were excluded from the group. Overall survival (OS) was a period defined as the time between the date of diagnosis and the date of death or the last follow‐up. Tumor tissues were embedded in paraffin for immunohistochemistry and frozen at −80 °C for RNA and protein extraction.

### Cell Culture and Reagents

Human breast cancer cell lines (MDA‐MB‐231, BT549, MDA‐MB‐468, MDA‐MB‐157, HCC1806 and SUM159PT), the normal mammary epithelial cell line MCF‐10A, and the HEK293T cell line were all purchased from the American Type Culture Collection (ATCC) and cultured according to the manufacturer's instructions. Chloroquine (CQ) and bafilomycin A1 (Baf‐A1) were purchased from MedChemExpress. Earle's Balanced Salt Solution (EBSS) was purchased from Beyotime Biotechnology. Cycloheximide (CHX) and proteasome inhibitor (MG132) were purchased from Yeasen.

### Bioinformatics Analyses

R package “DEseq2” was applied for differential gene expression analysis between normal breast tissues and TNBC samples with the criterion of |log2FC| > 1 and FDR < 0.05 in Gene Expression Omnibus database (GSE38959, GSE45827 and GSE65194).^[^ ^52^, ^53^ ^]^ Kaplan–Meier survival analyses for overall survival (OS) and recurrence‐free survival (RFS) were performed with log‐rank test p < 0.05 in TNBC patients from The Cancer Genome Atlas (TCGA) database. The divergence of markedly enriched pathways between two groups was ascertained by Gene Set Enrichment Analysis (GSEA).^[^ ^54^ ^]^

### Immunohistochemistry (IHC) and Hematoxylin‐eosin (H&E) Staining

For IHC staining of paraffin‐embedded tissues,^[^ ^55^ ^]^ sectioned slides were dewaxed in xylene and rehydrated through graded ethanol after baking at 65 °C for 2 h. Before incubation with primary antibody at 4 °C overnight and subsequently secondary antibody at room temperature for 1 h, antigen retrieval, blockage of endogenous peroxidase activity for 10 min and goat serum closure for 30 min were conducted successively. Then, slides were stained with hematoxylin after diaminobenzidine (DAB) substrate treatment. The antibodies used were: anti‐SAT1 (NB110‐41622, Novus Biologicals); anti‐vimentin (10366‐1‐AP, Proteintech); anti‐slug (12129‐1‐AP, Proteintech); anti‐N‐cadherin (13116, CST); anti‐LC3A/B (12741, CST); anti‐p62 (18420‐1‐AP, Proteintech). Semiquantitative analyses of H‐score was performed by multiplying the percentage of positive‐staining cells (1+ for ≤ 25%, 2+ for ≤ 50%, 3+ for ≤ 75%, and 4+ for ≤ 100%) by the staining intensity (0 for negative; 1+ for weak staining; 2+ for moderate staining and 3+ for strong staining). For H&E staining, stained sections were hematoxylin‐soaked for 3 min, washed with water for 30 min, stained with eosin for 3 min and covered with coverslips after dehydration in graded ethanol dilutions. All the represented images of stained slides were obtained from an automatic slide scanner.

### Transient Transfection and Stable Cell Line Construction

siRNAs and plasmids were transiently transfected into indicated cells with Lipofectamine 3000 Transfection Reagent (Invitrogen, USA), and total RNA and protein were harvested after 48 and 72 h respectively. For construction of stable cell lines, pLenti‐U6‐shRNA (SAT1)‐CBh‐Luc2‐tCMV‐mNeonGreen‐F2A‐Puro‐WPRE and pLV‐CMV‐mcherry‐P2A‐hYBX1‐HA‐IRES‐bla plasmids were generated to perform lentivirus production. After infection with lentivirus for 24 h, cells were cultured in fresh medium with puromycin (1 µg mL^−1^) or blasticidin S (5 µg mL^−1^) for 2 weeks, as indicated. Targeted sequences are listed in Table S1 (Supporting Information).

### RNA Isolation and Quantitative Real‐Time PCR (RT‐qPCR)

For indicated cells, total RNA was extracted using a quick RNA extraction kit (ES Science, RN001). Total RNA extraction from fresh or frozen tissues was carried out with TRIzol reagent (Invitrogen). Following reverse transcription, the detection of RNA expression levels was conducted by qPCR with a SYBR Green kit (Takara, RR420A) on the Bio‐Rad CFX96. Primer sequences are listed in Table S1 (Supporting Information).

### Western Blots and Antibodies

Total protein from cells and tissues was extracted using RIPA lysis buffer containing protease inhibitor (1:100). Standardization of protein concentration was performed with a BCA assay kit (Beyotime Biotechnology, China). After mixed with 5X SDS loading buffer and boiled at 95 °C for 10 min, each group of protein was loaded onto SDS‐PAGE gels for separation and subsequently transferred to a PVDF membrane (Millipore). Then, the membranes were incubated with primary targeted antibodies overnight at 4 °C. After washing, the membranes were incubated with the secondary antibody at room temperature for 1 h. Finally, immunoblotting was carried out using chemiluminescent reagents (Yeasen, China) following the manufacturer's instructions. Antibodies used included: anti‐SAT1 (61586, CST); anti‐YBX1 (20339‐1‐AP, Proteintech); anti‐β‐actin (AF7018, Affinity); anti‐vimentin (10366‐1‐AP, Proteintech); anti‐slug (12129‐1‐AP, Proteintech); anti‐ZEB1 (3396, CST); anti‐E‐cadherin (3195, CST); anti‐N‐cadherin (13116, CST); anti‐LC3A/B (12741, CST); anti‐p62 (18420‐1‐AP, Proteintech); anti‐IgG (30000‐0‐AP/B900620, Proteintech); anti‐Flag (66008‐4‐Ig/20543‐1‐AP, Proteintech), anti‐HA (51064‐2‐AP/66006‐2‐Ig, Proteintech); anti‐Myc (16286‐1‐AP, Proteintech); anti‐HERC5 (22692‐1‐AP, Proteintech); anti‐JUN (22114‐1‐AP, Proteintech); and anti‐mTOR (66888‐1‐Ig, Proteintech).

### Cell Proliferation Assays

For the CCK8 assay,^[^ ^56^ ^]^ cells were seeded at 2 × 10^3^ cells/well into 96‐well plates, and a mixture of fresh medium (90 µL) and CCK8 solution (10 µL) was added to each well on a given day. Absorbance at 450 nm was measured on a microplate reader after incubation at 37 °C for 2 h. For the colony formation assay,^[^ ^57^ ^]^ cells were seeded at 1 × 10^3^ cells/well in 6‐well plates and cultured for 14 days. After fixed with methanol for 20 min and stained with 0.1% crystal violet for 30 min, cell colonies were photographed.

### Cell Migration Assays

For transwell migration assay,^[^ ^58^ ^]^ cells were seeded at 2 × 10^4^ cells/well into 24‐well plates containing chambers with serum‐free medium in the upper layer and 20% serum medium in the lower layer. After incubation for 20 h, cells were fixed with methanol for 20 min, stained with 0.1% crystal violet for 30 min and photographed. For scratch wound healing assay,^[^ ^58^ ^]^ cells were plated in 6‐well plates and cultured until reaching 90% confluency. Three vertical scratches were created in each well and photographed. Cells continued to grow in serum‐free medium for 24 h and were re‐photographed. Images were processed through the Image J software.

### Co‐Immunoprecipitation (CoIP)

Protein was extracted from indicated cells in IP lysis buffer (Beyotime Biotechnology, China) containing the protease inhibitor PMSF (Beyotime Biotechnology, China). For each group, 80% of the total protein lysate was used as IP solution with the remaining 20% as input. After incubating with the primary antibody overnight at 4 °C, the IP solution was incubated with protein A/G magnetic beads (HY‐K0202, MCE) at 4 °C for another 3 h. The beads were detached by a magnetic holder, eluted in 1X SDS sample buffer, and boiled at 95 °C for 10 min.

In this study, CoIP assays were conducted in BT549 and SUM159PT cells, respectively. And two positive IgG controls and two experimental samples were further analyzed by mass spectrometry (MS) separately. The specific binding proteins of each experimental group were screened by excluding the proteins of the positive control group, and then the intersection of the two experimental groups were took to obtain candidate proteins.

### Immunofluorescence (IF)

After overnight incubation in 24‐well plates with internal crawlers, cells were fixed in 4% paraformaldehyde for 20 min, permeabilized with 0.3% Triton X‐100 (Invitrogen, USA) for 10 min, blocked with goat serum for 30 min, incubated with primary antibody overnight and fluorescent secondary antibody for 1 h at room temperature, stained with DAPI for 10 min and imaged by using Olympus fluorescence microscope (Olympus, Japan). Applied antibodies included anti‐SAT1 (10708‐1‐AP, Proteintech), anti‐YBX1 (sc‐398340, Santa Cruz), CoraLite488‐conjugated Goat Anti‐Mouse IgG (SA00013‐1, Proteintech) and CoraLite594‐conjugated Goat Anti‐Rabbit IgG (SA00013‐4, Proteintech).

### Chromatin Immunoprecipitation (ChIP)

The ChIP assay was conducted using a ChIP assay kit as directed (Bersin Bio, China). After collecting 2 × 10^7^ cells by centrifugation, DNA cross‐linking was performed with 1% formaldehyde at room temperature. The nuclear contents were isolated with lysis buffer from the washed precipitates. Then, the DNA segmented by ultrasound was further enriched by incubation with an antibody at 4 °C overnight. Next day, after inverse‐crosslinking at 65 °C overnight, the enriched DNA was extracted from the mixture and quantified using qPCR analysis. JUN antibody (22114‐1‐AP, Proteintech) was applied to complete this assay. The primer sequences for ChIP‐qPCR are also listed in Table S1 (Supporting Information).

### Dual‐Luciferase Reporter Assay

Cells were plated into 24‐well plates and co‐transfected with the corresponding plasmids for 48 h. After the cells were lysed, the activities of Renilla and Firefly luciferase were analyzed by a Dual Luciferase Reporter Assay Kit (Promega, USA) according to the manufacturer's instructions. Notably, plasmids of human SAT1 promoter wildtype and mutants were all generated with a pEZX‐FR01 vector (GeneCopeia, USA). The JUN overexpression plasmid was constructed with a pcDNA3.1(+) vector (Thermofisher, USA).^[^ ^59^ ^]^

### GFP‐ Monomeric Red Fluorescent Protein (mRFP)‐LC3 Adenoviral Transfection

BT‐549 cells infected with GFP‐mRFP‐LC3 adenoviral vectors (HanBio Technology, China) were cultured in DMEM with puromycin (1 µg mL^−1^) for 1 week. The cells were plated on glass‐bottomed dishes and incubated overnight. The autophagy flux was further observed with a confocal laser scanning microscope (LSM 880 with Airyscan; Zeiss, Dublin, CA, USA).^[^ ^60^ ^]^

### RNA Immunoprecipitation (RIP)

The RIP assay was performed by an RNA‐Binding Protein Immunoprecipitation Kit (Millipore, USA). Briefly, 2 × 10^7^ cells were collected with RIP lysis buffer containing protease inhibitor. The lysed cells were mixed with immunoprecipitation buffer, and rotated with primary or negative control antibodies at 4 °C overnight. The RNA‐protein complexes were extracted with magnetic beads, and enriched RNAs were purified by TRIzol (Invitrogen, CA, USA) for further qPCR analysis. Applied antibodies included anti‐YBX1 (20339‐1‐AP, Proteintech) and anti‐m5C (ab214727, Abcam).

### Dot Blot Assay

First, after denaturation by heating at 95 °C for 3 min and followed by cooling on ice immediately, quantitative mRNAs for each group were spotted onto Amersham Hybond‐N + membrane to optimize nucleic acid transfer for detection (GE Healthcare). Membranes were crosslinked under UV light and unconjugated RNAs were removed by washing with 1X PBST buffer. Before the membrane was incubated with anti‐m5C antibody (ab214727, Abcam) at 4 °C overnight, it was blocked with 5% nonfat milk in PBST for 1 h. The membrane was subsequently incubated with secondary antibody at room temperature for 1 h and the development of immunoblots was performed using chemiluminescent reagents (Yeasen, China). Similarly, the same mRNAs were spotted on the membrane for staining with 0.2% methylene blue in 0.3 m sodium acetate (pH 5.2).

### RNA Stability Assay

Cells were cultured in 12‐well plates overnight for treatment with actinomycin D (5 µg mL^−1^, Sigma) for 0, 4, 8, and 12 h. At indicated time points, total RNA was extracted and quantified for qPCR analysis to examine the alteration of mRNA half‐life.

### Animal Experiments

Four‐week‐old female BALB/c nude mice were purchased from Beijing Vital River Laboratory (Beijing, China). For xenograft model, luciferase‐labeled 1 × 10^7^ BT549 or 1 × 10^6^ SUM159PT cells with SAT1 knockdown stably were injected into the fat pads of mice mammary. Tumor growth was observed for 4 weeks and tumor volume was measured weekly. Tumors were visualized by injecting 150 mg kg^−1^ D‐luciferin potassium salt (ATT Bioquest) intraperitoneally in mice for in vivo bioluminescence imaging. At the end of the experiment, mice were sacrificed, and the xenografts were excised, photographed and deposited in 4% paraformaldehyde for subsequent tissue embedding. For the liver metastasis model, 2 × 10^6^ BT549 or SUM159PT cells with SAT1 knockdown stably were injected into the spleens of 6‐week‐old BALB/c female mice in a sterile surgical environment. The observation period was scheduled for 2 months, during which in vivo bioluminescence imaging was performed every two weeks to visualize the metastases. After the mice were sacrificed, livers were resected for further pathological evaluation. Each liver specimen was embedded and sectioned for HE staining to determine the number of metastatic nodules. All mice were reared in the animal laboratory of Sun Yat‐sen University Cancer Center under pathogen‐free conditions. Tumors larger than 15 mm in any dimension or heavier than 10% of the body weight were not admitted. Approval of all animal procedures was obtained from Institutional Animal Care and Use Committee of Sun Yat‐sen University Cancer Center.

### Statistical Analyses

All experimental assays were performed in triplicate and the results were presented as mean ± SD. Statistical analyses for all data were conducted with one‐way ANOVA, two‐way ANOVA, or Student's t‐test methods using GraphPad Prism software 8.0. The correlation analysis was achieved by Spearman test. Statistical significance was considered as p < 0.05 and denoted by asterisks (^*^ *p* < 0.05, ^**^ *p* < 0.01, ^***^ *p* < 0.001, and ^****^ *p* < 0.0001).

## Conflict of Interest

The authors declare no conflict of interest.

## Author Contributions

W.T., L.Z., Y.L., and Y.T. contributed equally to this work. F.Y., X.X., M.C., H.L., and W.T. designed the study. Y.T., L.Z., and Y.L. collected the data and performed statistical analyses. Y.Z., Q.T. and X.D. provided the clinical samples. K.C. and H.T. corrected the language writing. W.T. wrote the manuscript. F.Y., X.X., M.C., and H.L. finally reviewed the manuscript. All authors have approved the final manuscript.

## Supporting information

Supporting Information

Supplemental Table 1

## Data Availability

The data that support the findings of this study are available in the supplementary material of this article.
