# Supplementary material for: Autophagy Deficiency Induced by SAT1 Potentiates Tumor Progression in Triple‐Negative Breast Cancer
Source: Adv Sci (Weinh). 2024 Jul 29;11(36):2309903. doi: 10.1002/advs.202309903 (PMC11423137; doi:10.1002/advs.202309903)
Supplement: Supplementary file 1 — Supporting Information [file ADVS-11-2309903-s002.docx]

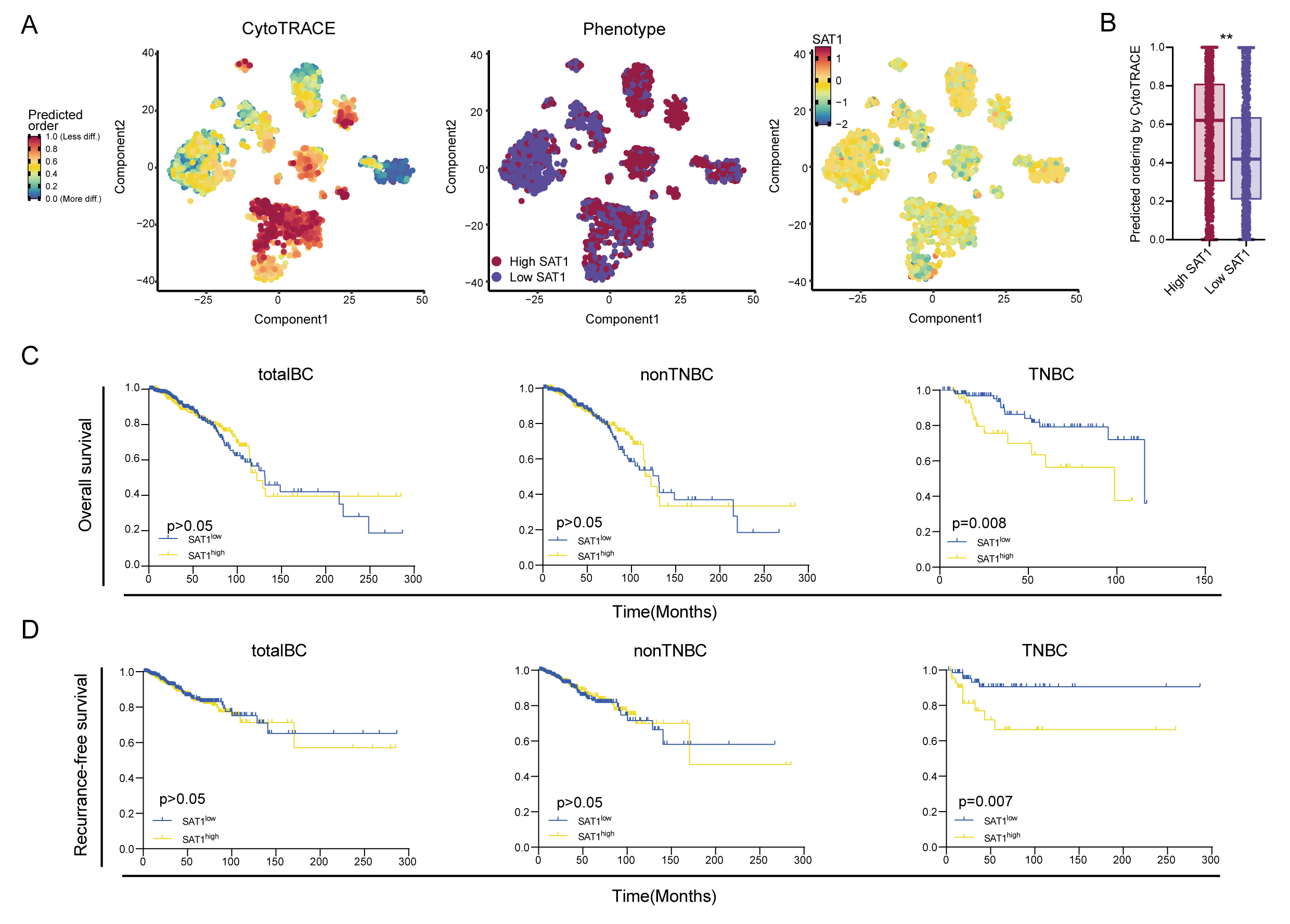


**Supplementary Figure S1.** High SAT1 expression indicates worse prognosis in TNBC. (A) The left tSNE plot depicted the distribution of CytoTRACE scores among cancer epithelial cells. Dark-blue indicates lower CytoTRACE scores (low stemness) while dark-red indicates higher CytoTRACE scores (high stemness). The middle and right tSNE plots labeled cancer epithelial cells by the SAT1 expression (High and low SAT1 expression were distinguished by the median value of SAT1 expression). (B) Box plots of relationship between CytoTRACE scores and expression levels of SAT1. (C-D) The Kaplan-Meier survival analyses for OS (C) and RFS (D) in breast cancer patients with different SAT1 expression levels among the TCGA-BRCA cohort (High and low SAT1 expression were distinguished by the median value of SAT1 expression).


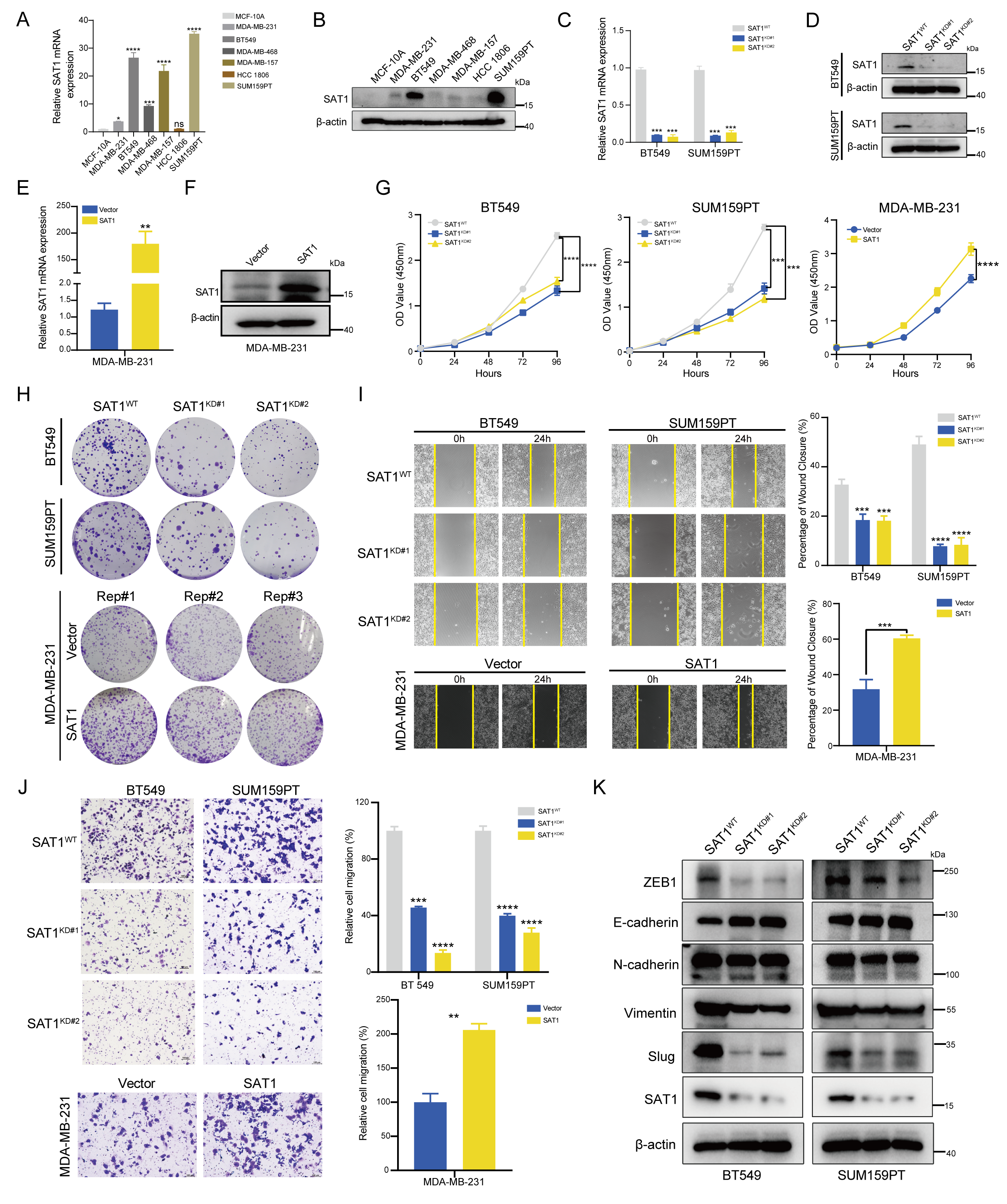


**Supplementary Figure S2.** SAT1 knockdown inhibits tumor malignancy of TNBC in vitro. (A-B) SAT1 upregulation was detected in various cell lines of TNBC compared to MCF-10A through RT-qPCR (A) and western blots (B). (C-D) The effectiveness of SAT1 knockdown in BT549 and SUM159PT cells was qualified by RT-qPCR (C) and western blots (D). (E-F) The effectiveness of ectopic overexpressed SAT1 in MDA-MB-231 cells was examined by RT-qPCR (E) and western blots (F). (G-H) The influence of SAT1 knockdown and overexpression on cell proliferation was detected by CCK8 (G) and clone formation assays (H). (I-J) The impact of SAT1 knockdown and overexpression on cell migration was determined with scratch wound healing (I) and transwell migration assays (J). (K) Western blots for EMT-related proteins to verify the involvement of SAT1 in epithelial-mesenchymal transition (EMT).


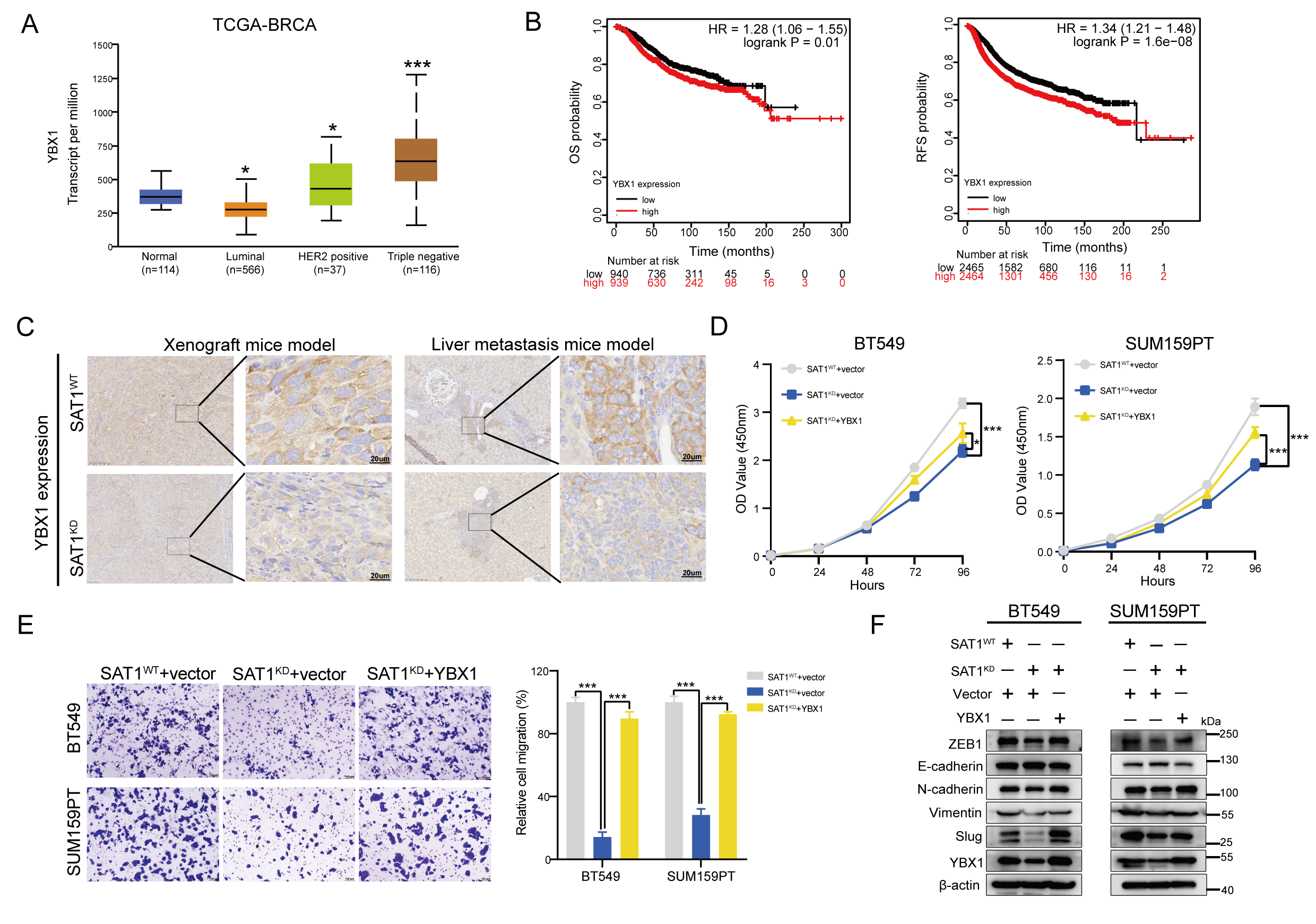


**Supplementary Figure S3.** SAT1 promotes TNBC progression through YBX1 mediation. (A) The expression of YBX1 in breast cancer patients from TCGA-BRCA cohort across subtypes. (B) The Kaplan-Meier survival analyses for OS and RFS in breast cancer patients with different YBX1 expression levels using Kaplan-Meier plotter. (C) Examination of YBX1 expression in tissues derived from mice xenograft models and liver metastasis models by IHC. (D) The rescue CCK8 assays to determine the proliferation ability of SAT1^KD^ cells in response to YBX1 overexpression. (E) The transwell migration assays to determine the migration ability of SAT1^KD^ cells in response to YBX1 overexpression. (F) Western blots for EMT markers in SAT1^KD^ cells in response to YBX1 overexpression.


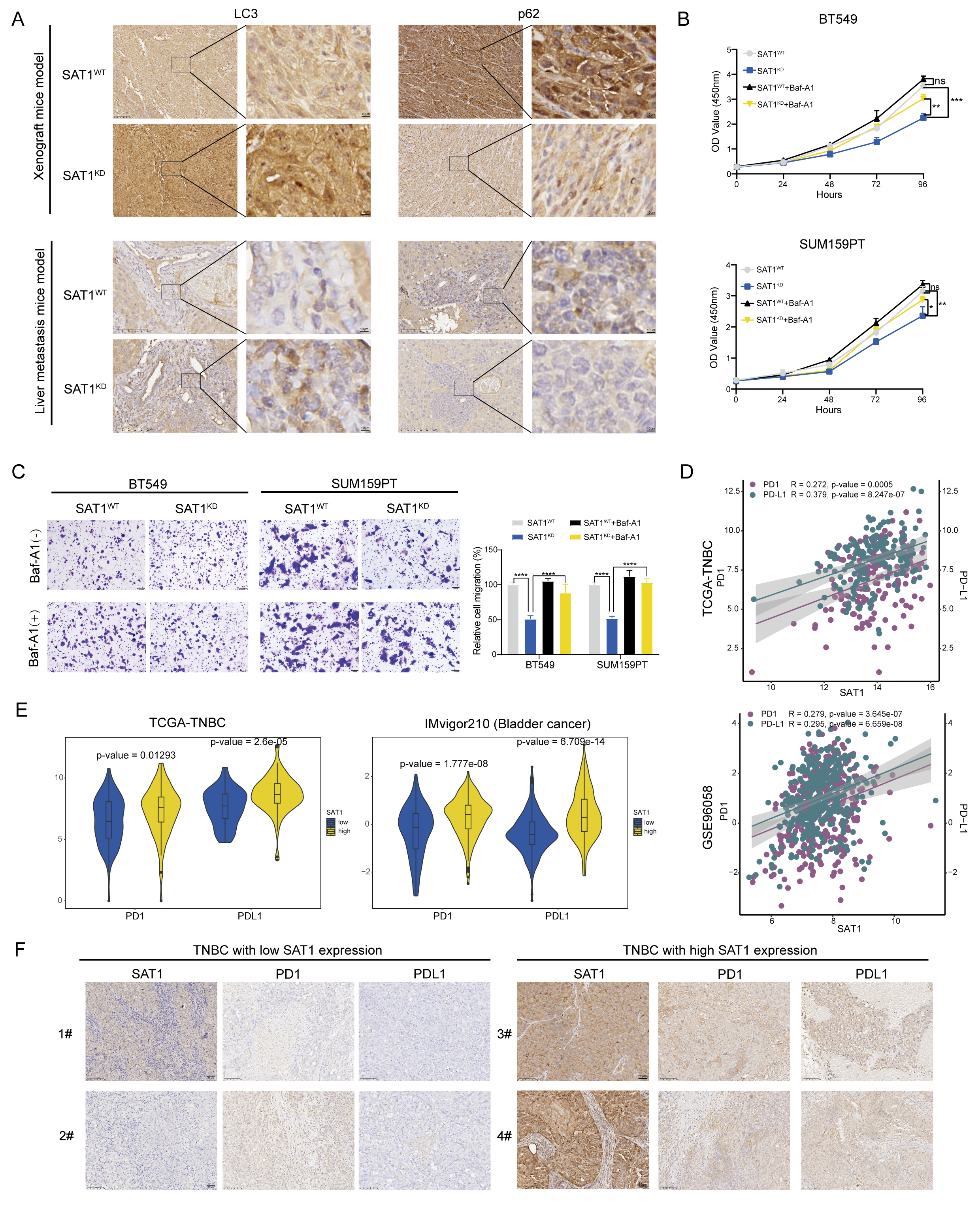


**Supplementary Figure S4.** Autophagy depression induced by SAT1 involves in TNBC advancement. (A) IHC staining for LC3 and p62 in primary tumors and liver metastasis tissues from mice models of both SAT1^WT^ and SAT1^KD^ groups. (B-C) The CCK8 proliferation assay (B) and transwell migration assay (C) were performed in SAT1^WT^ and SAT1^KD^ cells with or without BafA1 treatment. (D) The correlation analyses between SAT1 expression and immune checkpoints (PD1 and PD-L1) in TNBC samples of TCGA and GSE96058. (E) The expression boxplots in TCGA-TNBC and IMvigor210 datasets exhibited the relationship between SAT1 expression and immune checkpoints (PD1 and PD-L1). (F) The detection of SAT1, PD1 and PD-L1 expressions in TNBC tissues using IHC.
